# Associations Between Trail-Making Test Black and White Performance and Gray Matter Volume in Community-Dwelling Cognitively Healthy Adults Aged 40 to 80 Years

**DOI:** 10.3390/jcm14124041

**Published:** 2025-06-07

**Authors:** Chanda Simfukwe, Seong Soo A. An, Young Chul Youn

**Affiliations:** 1 Department of Bionano Technology, Gachon University, Seongnam-si 1342, Republic of Korea; chandaelizabeth94@gmail.com; 2 Department of Neurology, College of Medicine, Chung-Ang University, Seoul 06974, Republic of Korea; 3 Department of Medical Informatics, College of Medicine, Chung-Ang University, Seoul 06974, Republic of Korea

**Keywords:** cognitive aging, trail making test, executive function, magnetic resonance imaging, gray matter, thalamus, Asian people

## Abstract

**Background/Objective:** The Trail Making Test (TMT) is a widely used neuropsychological tool to assess processing speed (Part A) and executive function (Part B). However, the neuroanatomical substrates underlying its Black & White variant (TMT-B&W) and the influence of demographic factors remain poorly understood. This study aimed to identify gray matter (GM) correlates of TMT-B&W performance across unadjusted and covariate-adjusted models in cognitively healthy adults. **Methods:** In this cross-sectional study, 87 participants (40–80 years) underwent structural magnetic resonance imaging (MRI) and completed TMT-B&W. Whole-brain voxel-based morphometry (VBM) was conducted using FreeSurfer for preprocessing and Computational Anatomy Toolbox (CAT12)/Statistical Parametric Mapping (SPM12) for analysis. Two voxel-wise regression models (unadjusted and adjusted for age, education, gender, and total intracranial volume (TICV)) assessed GM associations with TMT-B&W-A-B performance. Statistical thresholds were voxel-level *p* < 0.001 (uncorrected) and cluster-level Family-Wise Error (FWE) correction (*p* < 0.001). **Results:** In unadjusted models, TMT-B&W-A performance correlated with GM reductions in the right orbitofrontal cortex (T = 42.64, equivk = 515.60, representing peak voxel level T-statistic and cluster size in voxels), while TMT-B&W-B linked to the right insular cortex (T = 50.65, equivk = 515.50). After adjustment, both tasks converged on the left thalamus (TMT-A: T = 8.05, equivk = 594; TMT-B: T = 8.11, equivk = 621), with TMT-B&W-B showing a denser thalamic cluster. Demographic covariates attenuated cortical associations, revealing thalamic integration as a shared mechanism. **Conclusions:** The thalamus emerges as a critical hub for TMT-B&W performance when accounting for demographic variation, while distinct cortical regions mediate task-specific demands in unadjusted models. These findings support the TMT-B&W as a practical, low-cost neurobehavioral marker of brain integrity in older populations.

## 1. Introduction

Cognitive aging is a growing global concern, as it is often accompanied by declines in memory, processing speed, executive function, and attentional control [1,2]. These changes not only affect quality of life but also increase the societal burden of age-related neurodegenerative diseases [3,4]. As populations age, identifying early structural markers of cognitive decline has become critical for timely intervention and risk prediction [5]. Neuropsychological tests, such as the Trail Making Test (TMT), the Clock Drawing Test (CDT), the Montreal Cognitive Assessment (MoCA), and the Mini-Mental State Examination (MMSE) are widely used to assess cognitive functioning. Yet, their neuroanatomical correlates, especially across culturally adapted versions, remain underexplored [6,7,8]. These tools were developed with a shared goal: to evaluate attention, mental flexibility, and visual scanning abilities in individuals with cognitive impairment [9]. Among them, the TMT is one of the most frequently utilized and is available in several versions, such as the Black & White TMT (TMT-B&W), the Color Trails Test, the circle-and-square version, and the original form involving the English alphabet [6]. The TMT typically requires individuals to sequentially connect randomly arranged numbers, letters, shapes, or colors [6,10]. It is traditionally composed of parts A and B, each designed to assess different aspects of cognitive function [10].

The TMT-B&W was developed in South Korea to minimize cultural and language-related biases as an alternative tool for assessing neurodegenerative conditions across diverse populations [10,11]. Instead of relying on the English alphabet, this version uses 15 numbered circles that are either black or white in background color [11]. In TMT-B&W Part A (TMT-B&W-A), odd numbers are presented in white circles and even numbers in black circles, with each number appearing only once, while in TMT-B&W Part B (TMT-B&W-B), there are two copies of the numbers 2–5, each displayed once in black and again in white. The number 1 is included only once, encircled in white [6,10,11]. This culturally neutral design enhances the test’s accuracy and applicability regardless of the participant’s linguistic or cultural background. Specifically, the TMTs are used to assess visual scanning, motor speed, attentional processes, and core cognitive functions that are particularly vulnerable to age-related decline [6]. Performance on the TMT is closely associated with a participant’s age and educational background, highlighting the importance of accounting for these factors when administering and interpreting TMT results [11,12].

There is increasing global interest in healthy sectors for early detection of age-related neurodegenerative diseases to prevent or delay their progression. Magnetic resonance imaging (MRI) has emerged as a valuable neuroimaging tool for evaluating brain structure and development due to its high soft-tissue contrast, multi-parametric capabilities, and non-invasive nature in cognitively healthy individuals [13,14]. However, the cognitive tasks typically used in MRI paradigms often do not align directly with standardized clinical neuropsychological assessments. This mismatch arises because neuropsychological tests cannot always be administered in the same standardized format within the imaging environment as in clinical settings. Consequently, relatively few brain mapping studies have incorporated established neuropsychological measures into their designs [15].

To address this methodological gap, the current study integrated MRI-based structural analyses using voxel-based morphometry (VBM) automated neuroimaging techniques that enable statistical patterns, whole-brain assessments of gray matter (GM) volume, and cortical structures [16,17,18]. Specifically, we examined associations between regional GM volume and performance on the culturally neutral TMT-B&W administered to cognitively healthy adults aged 40 to 80 years. Analyses controlled for age, gender, education, and total intracranial volume (TICV). The TICV is the total volume inside the skull, including brain tissue and cerebrospinal fluid. It is commonly used as a covariate in VBM to control for individual differences in head size and improve the accuracy of gray matter volume comparisons [18]. The TMT-B&W was selected for its sensitivity to core cognitive domains such as attention, visuomotor sequencing, and executive control functions that are particularly susceptible to age-related brain changes in frontal and parietal regions [6,10].

TMT-B&W Parts A and B are visuomotor tasks requiring rapid numeric sequencing and alternating attention as individuals connect numbers embedded within alternating black-and-white circles, engaging mental flexibility and visual scanning abilities [10,11]. Previous investigations into the neural correlations of TMT Trail A and B performance have consistently underscored the involvement of frontal brain regions in supporting task performance. For example, Kim et al. [10] demonstrated a robust association between TMT-B&W-A-B performance and frontal lobe cognitive assessments, affirming its validity in capturing executive and attentional processes. Similarly, Zakzanis et al. [19], using functional MRI (fMRI), identified significant frontal lobe activation during TMT Part A (TMT-A) execution, further highlighting its sensitivity to executive control mechanisms. Han et al. [20] also found that poorer performance on TMT-B&W, characterized by longer completion times and increased errors, was significantly associated with a more significant white matter hyperintensity (WMH) burden on MRI. These findings establish a strong theoretical and neurobiological foundation for using TMT-B&W-A-B as a meaningful proxy for assessing frontal lobe function in clinical and research settings.

In this study, we aimed to investigate the hypothesis that poorer performance on the TMT-B&W Trails A and B is associated with regional differences in GM volume, particularly within brain regions involved in attention, visuomotor integration, and executive function. Identifying these structural correlates in cognitively healthy adults may support the utility of TMT-B&W as a non-invasive, cost-effective behavioral marker for detecting subtle brain changes that precede clinical cognitive decline.

## 2. Materials and Methods

### 2.1. Study Design

This cross-sectional, observational study aimed to examine associations between cognitive performance on the TMT-B&W (Parts A and B) and GM volume. The primary independent variables were TMT-B&W completion times, while the primary dependent variable was regional GM volume, derived from anatomical preprocessing with FreeSurfer (version 7.3.2; surfer.nmr.mgh.harvard.edu). Secondary variables, including age, gender, education, and TICV, were included as covariates and adjusted in the VBM models to control for potential confounding effects.

### 2.2. Participants

This study enrolled a total of 87 participants (44 males and 43 females) who were cognitively and neurologically healthy. All participants were between 40 and 80 years old (Table 1) and deemed healthy according to the Christensen criteria [21]. Participants were recruited using consecutive sampling from a hospital-based community registry. No formal power calculation was performed; the sample size reflects the number of eligible individuals enrolled during the study period. The inclusion criteria were as follows: (1) >6 years of formal education; (2) a Mini-Mental State Examination (MMSE) score within one standard deviation of the age- and education-adjusted normative mean; (3) a score of ≤6 on the Korean Dementia Screening Questionnaire (KDSQ); and (4) a score of ≤7 on the short-form Geriatric Depression Scale (GDS). Exclusion criteria included a history of stroke, traumatic brain injury, major psychiatric illness, uncontrolled medical conditions, or MRI contraindications.

Participants completed a medical history review, MMSE, KDSQ assessment, and TMT-B&W-A-B and underwent an MRI. This cohort study was conducted at the hospital to assess the prevalence of cognitive impairments and the associated risk factors among the elderly in accordance with the Declaration of Helsinki guidelines. It was approved by the institutional review Board of Chung-Ang University Hospital (IRB No. 2009-005-19331) under ethics committee approval on 29 December 2020. Written informed consent was obtained from all participants prior to enrollment.

### 2.3. TMT-B&W

The TMT-B&W was administered in a standardized format, as described by Kim et al. [10]. The test sheet consisted of 25 encircled numbers, alternating between white numbers in black circles and black numbers in white circles. Participants were instructed to draw a continuous line connecting the numbers in ascending order, alternating between the two circle types (i.e., 1 [white] → 2 [black] → 3 [white] → 4 [black]…). The task was to be completed as quickly as possible, and the total completion time was recorded. In addition to completion time, we collected composite timing variables, B-A, B/A, and error counts for Parts A and B (Table 1).

The TMT-B&W has been psychometrically validated in Korean populations, demonstrating high reliability and sensitivity to executive dysfunction and aging-related cognitive decline [6,10,11]. Normative interpretation in this study was based on a sample of cognitively healthy Korean adults aged 40–80 years, supporting the test’s applicability as a culturally neutral alternative to the conventional TMT. This tool minimizes linguistic and educational confounds, making it suitable for diverse populations [10]. The test was administered in a quiet room, with standardized instructions provided verbally by a trained examiner. Participants completed Part A before Part B and were allowed to ask questions before starting each section.

### 2.4. Brain MRI

Structural brain MRI was conducted using a 3.0 Tesla scanner (Philips Intera Achieve, Amsterdam, The Netherlands) equipped with an 8-channel head coil. Imaging protocols included high-resolution three-dimensional T1-weighted sequences, fluid-attenuated inversion recovery (FLAIR), T2-weighted, and susceptibility-weighted imaging (SWI).

### 2.5. Imaging Preprocessing

Structural brain MRI scans for all participants were preprocessed using FreeSurfer, a widely validated software package for the automated analysis and reconstruction of cortical and subcortical brain structures. FreeSurfer supports multiple imaging modalities, including structural MRI, functional MRI, diffusion tensor imaging (DTI), and positron emission tomography (PET) and is extensively used in neuroimaging research for cortical surface modeling and volumetric segmentation [18].

In this study, the “recon-all” command-line pipeline was employed for the full cortical reconstruction and volumetric segmentation process. This procedure includes several critical steps, such as motion correction, non-uniform intensity normalization, skull stripping, gray/white matter boundary detection, automated topology correction, and parcellation of the cerebral cortex into standard anatomical regions. Specifically, subcortical GM segmentation was performed as part of this pipeline to generate participant-specific brain volume data, which served as the basis for downstream VBM analyses. All preprocessing steps were executed in a standardized computing environment using Ubuntu Linux OS version 22.04 LTS (ubuntu.com, accessed on 8 March 2025).

### 2.6. Data Analysis and VBM

After preprocessing and GM volume extraction, VBM analysis was conducted using the Computational Anatomy Toolbox (CAT12) (neuro-jena.github.io, accessed on20 February 2025), an extension within Statistical Parametric Mapping (SPM12) (www.fil.ion.ucl.ac.uk, accessed on 8 March 2025), running on MATLAB version R2020b (www.mathworks.com, accessed on 8 March 2025). The GM images were spatially normalized to Montreal Neurological Institute (MNI) space using CAT12’s high-dimensional registration framework, which applies geodesic shooting for precise inter-subject alignment. Modulation was performed using Jacobian determinants derived from the normalization process to preserve the original tissue volume [17]. The resulting modulated GM maps were then smoothed with an isotropic Gaussian kernel of 12 mm full width at half maximum (FWHM) to reduce noise and account for inter-subject anatomical variability, in line with the assumptions of Gaussian random field theory. The MNI avg152T1 template, derived from a healthy young adult cohort, was used as the standard anatomical reference for normalization and result visualization. To improve segmentation accuracy in the older adult sample, age-adapted tissue probability maps provided by CAT12 were applied during preprocessing.

The association between GM volume and cognitive performance on the TMT-B&W was assessed using two separate voxel-wise regression models implemented within the general linear model framework in SPM12. Performance scores from TMT-B&W Parts A and B were entered as independent variables in each respective model (unadjusted model). To control for potential confounding factors, age, education, gender, and TICV were included as covariates of no interest (adjusted model). This two-tiered approach is widely used in neuroimaging studies to distinguish associations that are potentially confounded by demographic variables from those that remain statistically robust under adjustment [22,23]. The TICV was estimated using the CAT12 implemented in SPM12, which provides automated, reproducible, and rater-independent measurements of intracranial volume. TICV included brain tissue, cerebrospinal fluid, and vasculature and was included to account for individual differences in head size [18,24].

Statistical thresholds were set at *p* < 0.001 (uncorrected) at the voxel-level and cluster-level Family-Wise Error (FWE) correlation at *p* < 0.001, with an extent threshold of 20 voxels (2 × 2 × 2 mm^3^ resolution). These models provided voxel-wise estimates of the relationship between TMT-B&W performance and regional GM volume while adjusting for covariates. In addition to voxel-level statistics, regression coefficients and associated statistics for covariates included in the adjusted models were extracted and summarized to assess their contributions. To visualize the results, the xjview (www.alivelearn.net/xjview/, accessed on 8 March 2025) graphical user interface (GUI) was used on the MNI avg152T1 template.

## 3. Results

A total of 87 cognitively healthy participants were included in the study. The mean age was 62.49 ± 7.36 years (mean ± standard deviation). The average MMSE score was 28.3 ± 1.38, and the mean KDSQ score was also 2.72 ± 1.45. Participants had an average of 10.34 ± 3.41 years of formal education (Table 1). Descriptive statistics for performance on TMT-B&W-A-B are presented in Table 1.

The whole-brain VBM identified significant GM correlates of performance on both Part A and Part B of the TMT-B&W. Analyses were performed across progressive models controlling for demographic covariates, including age, education, gender, and TICV. In the unadjusted base model, TMT-B&W-A performance, primarily indexing processing speed and visual search, revealed a similarly large cluster in the right orbitofrontal cortex (T = 42.64; equivk = 515.60; MNI: 19.5, 19.5, −13.5, representing peak voxel level T-statistic and cluster size in voxels), consistent with broad prefrontal involvement in sequencing tasks. In contrast, TMT-B&W-B performance reflective of executive functioning was associated with a larger cluster in the right insular cortex (T = 50.65; equivk = 515.50; MNI: 19.5, −24, −7.5; *p* (FEW) < 0.001) (Table 2), implicating core regions of the salience and executive control networks.

The fully adjusted model (age, education, gender, and TICV), for both TMT-B&W-A and TMT-B&W-B, revealed convergent associations in the left thalamus (TMT-A: T = 8.05; equivk = 594, MNI: −19.5, 25.5, −4.5; TMT-B: T = 8.11; equivk = 621; −19.5, 25.5, −4.5), suggesting that subcortical structures may serve as standard integrative hubs for task execution when accounting for demographic variation. Supplementary Materials (Tables and Figures) provides detailed regression coefficients, standard errors, t-statistics, and *p*-values for all covariates in the adjusted voxel-wise linear models. In addition, diagnostic evaluations, including residuals versus fitted value plots, quantile–quantile (Q-Q) plots, variance inflation factors, and model fit indices (R-squared, F-statistics) demonstrate that the assumptions of linear regression are reasonably met, supporting its application in the analysis of TMT-B&W performance.

**Figure 1:**
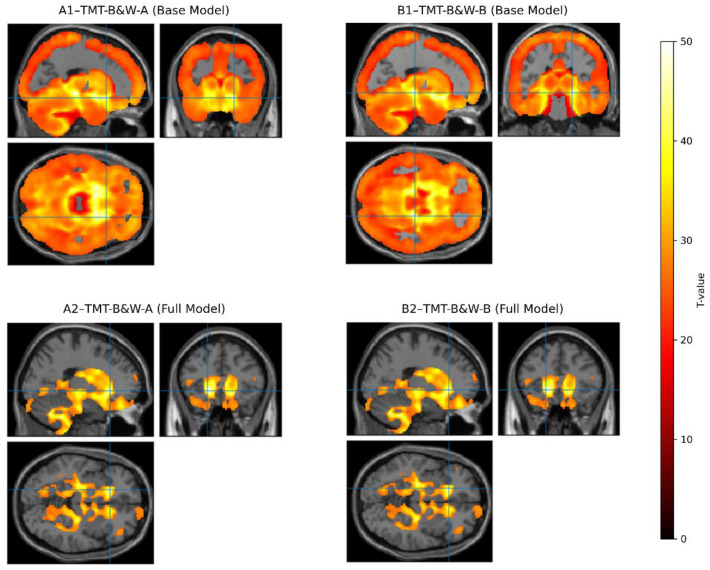
Gray matter associations with TMT-B&W-A and TMT-B&W-B performance across the base and fully adjusted models. Abbreviations: TMT-B&W-A = Trail-Making Test-Black And White Part A; TMT-B&W-B = Trail-Making Test-Black And White Part B; Base model = unadjusted; Full model = adjusted education, gender, and TICV; FEW = Family-Wise Error; MNI = Montreal Neurological Institute coordinate system. Note: Panels A1 and A2 display clusters associated with TMT-B&W-A, while panels B1 and B2 correspond to TMT-B&W-B. The top row (A1, B1) illustrates results from the base (unadjusted) model, and the bottom row (A2, B2) depicts fully adjusted models (controlling for age, education, gender, and TICV). All clusters are thresholded at *T* > 3.1 (FWE corrected *p* < 0.001) and overlaid on the MNI avg152T1 template using xjview software. The colorbar ranges from 0 to 50 to accommodate the high T-scores in the base models (A1/B1), ensuring values are not truncated. A mouse cursor highlights the peak MNI coordinates [x, y, z] (Table 2) at the thalamic convergence site in the model, emphasizing spatial specificity; Blue crosshairs indicate the center coordinates of the statistical activation map in each anatomical view.

The VBM analyses revealed distinct GM correlates for TMT-B&W-A and TMT-B&W-B performance in both unadjusted (base) and fully adjusted models (controlling for age, education, gender, and TICV). In the base model, TMT-B&W-A performance was negatively associated with GM volume in a large cluster spanning the right orbitofrontal cortex (T = 42.64; equivk = 515.60) (Figure 1), consistent with its role in attentional sequencing and visual processing. In contrast, the TMT-B&W-B base model identified a right insular cortex cluster (T = 50.65; equivk = 515.50), aligning with salience and executive control demands specific to cognitive flexibility.

After covariate adjustment, both models converged on a shared subcortical region, the left thalamus, albeit with nuanced spatial differences. In the fully adjusted model, TMT-B&W-A showed a distributed thalamic cluster (T= 8.05; equivk= 594), while TMT-B&W-B exhibited a denser and more focal thalamic association (T = 8.11; equivk = 621). This reduction in cortical extent alongside subcortical convergence emphasizes the distinct cortical pathways supporting each task while also suggesting shared integrative thalamic mechanisms under demographic control.

## 4. Discussion

In this study, we investigated the relationship between GM volume and timed performance on the TMT-B&W in a cohort of cognitively healthy adults using VBM. In the unadjusted models, distinct cortical patterns emerged: the right orbitofrontal cortex was significantly associated with TMT-B&W-A performance, while the right insular cortex showed strong correlations with TMT-B&W-B. These findings are consistent with the established roles of the orbitofrontal cortex in attentional sequencing and the insula in salience detection and cognitive control [25,26,27]. The inclusion of both unadjusted and adjusted models allows us to identify candidate regions and determine which associations remained robust after controlling for demographic covariates, consistent with best practices in neuroimaging studies.

After controlling for age, education, gender, and TICV, both tasks demonstrated convergent associations in the left thalamus. Interestingly, TMT-B&W-B exhibited a more focal thalamic cluster, whereas TMT-B&W-A showed a broader spatial pattern. This shift from cortical to subcortical regions following covariate adjustment highlights the thalamus’s integrative role in executive and attentional processes and underscores how demographic factors modulate observed brain–behavior associations [28,29,30].

VBM offers a robust analysis technique used to investigate brain–behavior relationships in the context of neuropsychological function. Developed to enhance the sensitivity of MRI in detecting focal anatomical differences, VBM is implemented within the SPM12 framework [16,17]. It involves segmenting T1-weighted MRI scans into gray and white matter probability maps, which are then spatially normalized to a standard template, allowing for voxel-wise comparisons of GM volume across individuals or groups [17]. In addition to VBM, automated neuroimaging pipelines such as FreeSurfer are commonly used to extract cortical and subcortical brain volumes and surface-based measures, providing complementary structural information for morphometric analyses [18]. A significant advantage of whole-brain VBM over traditional rater-based methods, such as manually tracing anatomically defined regions of interest (ROIs), is that it eliminates the need for prior assumptions about brain regions’ location, size, or shape. Manually defined ROIs often rely on anatomical landmarks such as sulci or tissue boundaries [18,19], but they may inaccurately include or exclude relevant areas, potentially reducing sensitivity and precision. VBM enables comprehensive and unbiased assessment of the entire brain or targeted regions with improved spatial resolution, making it a practical approach for examining structural brain correlates of cognitive performance [17].

Previous studies have reported mixed findings regarding frontal lobe volume and executive function performance, particularly on TMT variants [31,32,33]. Many of these inconsistencies likely stem from methodological differences, including the use of ROI-based analyses and the inclusion of clinical populations. In contrast, our use of whole-brain, data-driven VBM in a demographically controlled, non-clinical sample revealed robust associations between GM volume and cognitive performance. This approach captured subtle structural variations that may be missed in region-restricted methods, particularly when exploring task-specific constructs such as cognitive flexibility (TMT-B&W-B) and visual scanning (TMT-B&W-A) [34,35].

Our observation of thalamic convergence in the fully adjusted models aligns with previous work identifying the thalamus as a subcortical hub for relaying and integrating cortical signals during executive function tasks [36,37]. These findings suggest that while cortical regions may contribute more prominently in unadjusted models, subcortical structures like the thalamus become more apparent when controlling for key demographic confounders. This is particularly relevant in aging populations, where cortical thinning and educational disparities may mask subcortical contributions in simpler models [38,39]. Importantly, the predominance of frontal effects in unadjusted models does not imply exclusive involvement of these regions. Prior functional imaging research has highlighted distributed neural networks underpinning TMT performance, including parietal and cerebellar regions [32]. However, the convergence of both TMT-B&W-A and B on the thalamus in adjusted models suggests shared integrative pathways supporting task execution under demographic control.

Although the TMT-B&W shares core cognitive demands with the conventional TMT, its culturally neutral design, utilizing black and white circles instead of alphanumeric characters, minimizes linguistic and educational confounds [6,11]. This is particularly important in non-Western and aging populations, including the Korean cohort examined in this study, where standard TMT variants may conflate executive function with symbolic literacy or cultural familiarity [6,10]. Specifically, alphanumeric sequencing in TMT-B may disproportionately engage frontal regions associated with learned symbolic processing [40,41], potentially obscuring subcortical contributions. In contrast, the TMT-B&W enables a more direct assessment of domain-general processes, such as visual scanning, processing speed, and cognitive flexibility, independent of culturally acquired skills [6,10,12]. Notably, the thalamic and frontal GM associations observed in our study remained robust after adjusting for age, education, gender, and TICV, supporting their interpretation as core neural substrates rather than sociocultural artifacts, with TICV adjustment accounting for individual differences in head size [42]. While prior studies have reported similar regions using conventional TMT [19,22], our findings demonstrate that these associations persist under culturally debiased conditions, reinforcing their generalizability.

Despite the high spatial resolution and robustness of our neuroanatomical analyses, the present findings are inherently correlational and should not be interpreted as establishing causality or definitive neural substrates for TMT-B&W performance. The inclusion of only cognitively healthy older adults likely constrained variability in both neuropsychological function and GM volume, thereby limiting the generalizability of these results to broader or clinical populations. To enhance applicability and deepen understanding of the structural underpinnings of cognitive performance, future research should include more diverse cohorts, such as individuals with cognitive impairments or at-risk populations. The relatively modest sample size (N = 87) in the present study, while representative of a well-characterized and demographically controlled cohort, may limit statistical power and generalizability. However, the robustness of the observed GM associations using the culturally debiased TMT-B&W underscores the validity of our findings within this population. Prior VBM research has shown that stable brain–behavior associations can be identified with modest sample sizes, provided that appropriate statistical controls are applied. Lorca-Puls et al. (2018) reported reliable effects with samples near N = 90, while Scarpazza et al. (2015) identified a median sample size of 47 across 324 studies, reflecting prevailing norms in the field [43,44].

Although our models accounted for critical demographic covariates, namely age, education, gender, and TICV, additional factors such as MMSE and KDSQ were not explicitly included in the statistical models. Furthermore, latent or preclinical neurodegenerative changes, which are not readily detectable in cognitively healthy populations, may have influenced the observed associations. To enhance the robustness and interpretability of structural–cognitive relationships, future studies should incorporate longitudinal designs and integrate a broader array of cognitive, clinical, and biological measures. Moreover, the inclusion of complementary ROI and surface-based approaches may enhance regional specificity and anatomical resolution. This has been acknowledged in the present study as an important direction for future validation efforts.

The present findings must be interpreted in light of certain methodological limitations. First, the neuropsychological measures used, particularly TMT-B&W, are cognitively multifaceted, engaging in a range of overlapping domains such as attention, visuospatial processing, and executive function, which may complicate interpretations of regional specificity. Additionally, the neuroimaging data were derived from static structural T1-weighted scans, which, while optimal for GM segmentation, are not sensitive to other clinically relevant pathologies such as microvascular lesions or metabolic dysfunction. Structural contrast alone may not fully capture the functional or physiological integrity of brain tissue. Furthermore, spatial normalization using the MNI avg152T1 template derived from young adult brains may introduce registration and segmentation bias due to the older age of our sample. Morphological changes, such as cortical atrophy, may affect alignment accuracy. To mitigate this, we applied age-adapted tissue probability maps and conducted rigorous quality control. Although MNI152-based templates are commonly used in aging studies [45,46,47], future research may benefit from age-matched or population-specific templates to enhance anatomical precision.

Voxel-wise regression models are widely used in VBM due to their robustness, interpretability, and compatibility with the SPM framework [48,49]. These models have also demonstrated adequate sensitivity to unbiased brain changes, even under linear assumptions [49,50]. Moreover, VBM necessitates normalization to a standard template, a process that may be less precise in individuals with subtle atrophy, even when subject-specific templates are employed. These transformations could introduce minor spatial inaccuracies, particularly in aging populations. While our statistical models accounted for age linearly, nonlinear age-related effects on brain structure and cognition may not have been fully captured. Future investigations incorporating multimodal imaging, nonlinear modeling, and broader risk profiling, including genetic, cardiovascular, and environmental variables, are warranted to clarify the structural substrates that underline cognitive performance across the aging spectrum. Despite these constraints, the current results offer a valuable whole-brain map of GM correlates linked to TMT-B&W performance in a cognitively healthy cohort, providing insight into structural markers of attention and executive function in normal aging.

These findings highlight the potential of the TMT-B&W, in combination with structural MRI, as a culturally neutral and low-cost tool for detecting early brain changes associated with cognitive aging. Its sensitivity to both frontal and thalamic GM variations supports its clinical utility in diverse and resource-limited settings. Future research should validate these findings in clinical populations, employ longitudinal designs to assess predictive value, and incorporate multimodal imaging to elucidate underlying neural mechanisms.

## 5. Conclusions

This study delineates the neuroanatomical substrates underlying TMT-B&W performance in cognitively healthy adults, revealing distinct cortical correlates and shared subcortical involvement. In unadjusted models, TMT-B&W-A was associated with GM volume in the right orbitofrontal cortex, while TMT-B&W-B was linked to the right insular cortex regions consistent with visual sequencing and salience processing. After adjusting for age, education, gender, and TICV, these effects shifted toward a convergent subcortical cluster in the left thalamus, suggesting a compensatory role of thalamic structures in cognitive processing. These findings emphasize the critical influence of demographic and cultural factors on brain–behavior relationships and support the utility of the TMT-B&W as a low-cost, culturally unbiased, and sensitive marker of age-related structural variation. While cross-sectional and correlational, the study adds to growing evidence implicating the thalamus in higher-order cognitive integration. Longitudinal and multimodal approaches are needed to further elucidate these associations, particularly in at-risk or clinically vulnerable populations.

## Figures and Tables

**Table 1:** Summary statistics of participant’s characteristics and TMT-B&W performance.

Variable	Mean ± SD	Range	n (%)
**Demographics**			
Age (years)	62.49 ± 7.36	40–80	-
Education (years)	10.34 ± 3.41	3–18	-
Gender—Male	-	-	44 (50.6%)
Gender—Female	-	-	43 (49.4%)
MMSE Score	28.13 ± 1.38	23–30	-
KDSQ Score	2.72 ± 1.45	0–6	-
**TMT-B&W Performance**			
TOTAL_A (s)	82.41 ± 43.49	23.35–254.54	-
TOTAL_B (s)	194.31 ± 128.30	56.80–840.56	-
SUB_AB (s)	111.91 ± 108.71	8.55–674.31	-
DIV_AB (ratio)	2.49 ± 1.14	1.04–6.69	-
MISS_A (errors)	1.16 ± 1.62	0–8	-
MISS_B (errors)	2.98 ± 3.96	0–16	-

Abbreviations: SD = standard deviation; n (%) = number of participants and percentage of total sample; KDSQ = Korean Dementia Screening Questionnaire; MMSE = Mini-Mental State Examination. Note: TOTAL_A, total time (in seconds) to complete TMT-B&W Part A; TOTAL_B, total time (in seconds) to complete TMT-B&W Part B; SUB_AB, difference between TOTAL_B and TOTAL_A, representing additional time required for executive switching demands; DIV_AB, ratio of TOTAL_B to TOTAL_A, indicating relative executive cost; MISS_A, number of errors made during Part A (e.g., sequencing or skipping errors); MISS_B, number of errors (mistakes) made during Part B, reflecting accuracy under complex switching demands; Bold text indicates subsection headers for grouped variables.

**Table 2:** Significant gray matter clusters associated with TMT-B&W performance under varying covariate models.

Model	Peak MNI (x, y, z)	Cluster Size (Equivk)	Peak T	Anatomical Region
**TMT-B&W-A**				
Base	19.5, 19.5, −13.50	515.60	42.64	Right Orbitofrontal Cortex *
+ Age + Edu + **gender** + **TICV**	−19.5, 25.5, −4.50	594	8.05	Left Thalamus *
**TMT-B&W-B**				
Base	19.5, −24, −7.50	515.50	50.65	Right Insular Cortex *
+ Age + Edu + **gender** + **TICV**	−19.5, 25.5, −4.50	621	8.11	Left Thalamus *

Abbreviations: TMT-B&W-A = Trail Making Test Black & White Part A; TMT-B&W-B = Trail Making Test Black & White Part B; MNI = Montreal Neurological Institute coordinate system; equivk = Cluster size, expressed as the number of contiguous voxels; Peak T = Maximum T-score observed within the cluster, FEW = Family-Wise Error correction; Edu = Education; Gender = Biological gender; TICV = total intracranial volume; Base = unadjusted; Adjusted (age, education, gender, TICV). Note: * Clusters were thresholded at a voxel-level significance of *p* < 0.001 (uncorrected) and survived cluster-level FWE correction at *p* < 0.001. T-scores reflect peak voxel statistics within each cluster. MNI coordinates (x, y, z) are reported in millimeters within the Montreal Neurological Institute standard space, derived from a 1.5 mm isotropic voxel grid.

## Data Availability

The data supporting the findings of this study are available upon reasonable request from the corresponding author Y.C.Y via email. Due to confidentiality agreements and institutional policies protecting sensitive patient information from the hospital where the study was conducted, the data cannot be made publicly available. Requests will be evaluated to ensure compliance with ethical and legal standards.

## Supplementary Materials

The following supporting information can be downloaded at: https://www.mdpi.com/article/10.3390/jcm14124041/s1, Figure S1: Residuals vs. Fitted Plot for TMT-B&W-A (Base Model). Figure S2: Q-Q Plot of Residuals for TMT-B&W-A (Base Model). Figure S3: Residuals vs. Fitted Plot for TMT-B&W-B (Base Model). Figure S4: Q-Q Plot of Residuals for TMT-B&W-B (Base Model). Figure S5: Residuals vs. Fitted Plot for TMT-B&W-A (Adjusted Model). Figure S6: Q-Q Plot of Residuals for TMT-B&W-A (Adjusted Model). Figure S7: Residuals vs. Fitted Plot for TMT-B&W-B (Adjusted Model). Figure S8: Q-Q Plot of Residuals for TMT-B&W-B (Adjusted Model). Table S1: Covariate Statistics from Adjusted Regression Models for TMT-B&W Performance. Table S2: Summary of Model Fit for TMT-B&W Regression Models. Table S3: Variance Inflation Factors.
