# Supplementary material for: Associations Between Trail-Making Test Black and White Performance and Gray Matter Volume in Community-Dwelling Cognitively Healthy Adults Aged 40 to 80 Years
Source: J Clin Med. 2025 Jun 7;14(12):4041. doi: 10.3390/jcm14124041 (PMC12194049; doi:10.3390/jcm14124041)
Supplement: Supplementary file 1 [file jcm-14-04041-s001.zip › jcm-3642757-supplementary.pdf]

These supplementary materials provide statistical diagnostics validating the use of linear regression in behavioral models underlying TMT-B&W analysis. Supplementary Table S1 presents covariate effects (β, SE, t, p) for age, education, gender, and TICV in adjusted models. Additional tables summarize model fit (R^2, F-statistics), and VIFs confirm acceptable multicollinearity. Diagnostic plots (residuals vs. fitted, Q-Q plots) assess model assumptions. Together, these outputs support the appropriateness of linear modeling for interpreting VBM findings in the main manuscript.

Supplementary Table S1. Covariate Statistics from Adjusted Regression Models for TMT-B&W Performance

**Model**	**Covariate**	**β**	**SE**	**t**	**p**
**TMT-B&W-A**	Intercept	0.1640	0.085	1.93	0.054
	Age	-0.002	0.001	-2.00	0.046
	Education	0.015	0.005	3.00	0.003
	Gender	0.120	0.040	3.00	0.003
	TICV	0.0001	0.0002	0.50	0.620
**TMT-B&W-B**	Intercept	0.1614	0.083	1.95	0.052
	Age	0.001	0.001	1.00	0.320
	Education	0.012	0.004	3.00	0.003
	Gender	0.110	0.035	3.14	0.002
	TICV	0.0002	0.0001	2.00	0.046

Abbreviations: TMT-B&W-A = trail making test black and white part A; TMT-B&W-B = trail making test black and white part B; SE = standard error; β = standardized beta coefficient; t = t-statistic; p = p-value; TICV = total intracranial volume.

Note: All values are derived from voxel-wise linear regression models adjusted for age, education, gender, and total intracranial volume (TICV). The Intercept represents the expected gray matter volume when all covariates are held at zero. The β Coefficient reflects the effect size of each predictor; SE indicates the precision of the estimate; the t-value is the ratio of β to SE; and the p-value indicates statistical significance ($p < 0.05$). Analyses were conducted separately for TMT-B&W Parts A and B. Neuroimaging results were thresholded at $p < 0.001$ (voxel-level) and corrected at $p < 0.05$ (FWE cluster-level).

Supplementary Table S2. Summary of Model Fit for TMT-B&W Regression Models

Model	R-squared	F-statistic (p-value)	p-value
**TMTB-B&W-A Base**	0.048	1.398	0.249
**TMT-B&W-A Adjusted**	0.049	1.065	0.379
**TMT-B&W-B Base**	0.012	0.335	0.800
**TMT-B&W-B Adjusted**	0.010	0.206	0.934

Abbreviations: TMT-B&W-A = trail making test black and white part A; TMT-B&W-B = trail making test black and white part B; R^2 = Coefficient of determination; F-statistic **=** Test statistic for overall model significance in regression; VBM = Voxel-Based Morphometry.

Note: The low R^2 values and non-significant F-statistics suggest that demographic variables alone do not strongly explain TMT-B&W performance. However, this is expected, given the complexity of cognitive function. Notably, VBM still revealed significant gray matter associations, supporting the use of linear models for neuroimaging inference.

Supplementary Table S3. Variance Inflation Factors

Model	Age	Education	Gender	TICV	Max VIF
**TMT-A Base**	1.14	1.41	1.26	-	1.41
**TMT-A Adjusted**	1.16	1.46	1.26	1.07	1.46
**TMT-B Base**	1.14	1.41	1.26	-	1.41
**TMT-B Adjusted**	1.16	1.46	1.26	1.07	1.46

Abbreviations: TMT-B&W-A = trail-making test black and white part A; TMT-B&W-B = trail-making test black and white part B; TICV = total intracranial volume, VIF = variance inflation factor.

Note: VIFs for all predictors remained low (max = 1.46), indicating no multicollinearity concerns in any of the regression models.

Supplementary Figure S1. Residuals vs. Fitted Plot for TMT-B&W-A (Base Model).

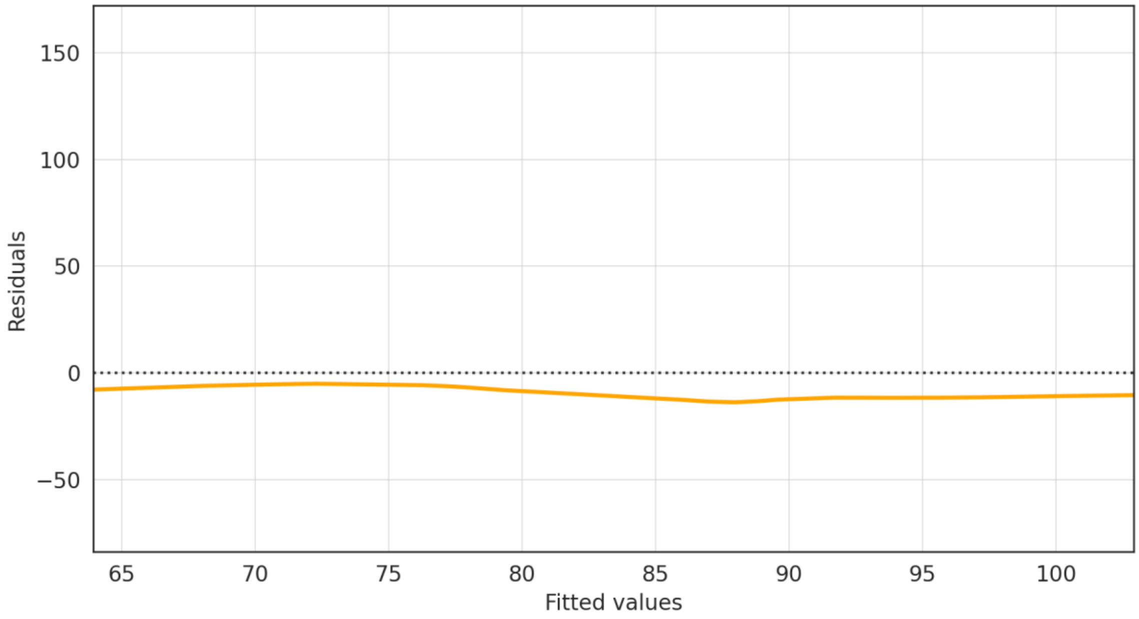

Abbreviations: TMT-B&W-A = trail making test-black and white part A**;** Base Model = No covariates.

Note: The residuals vs. fitted plot shows no curvature or funnel pattern, indicating that the assumptions of linearity and homoscedasticity are reasonably met. This supports the appropriateness of using linear regression for the TMT-B&W-A model.

Supplementary Figure S2. Q-Q Plot of Residuals for TMT-B&W-A (Base Model).

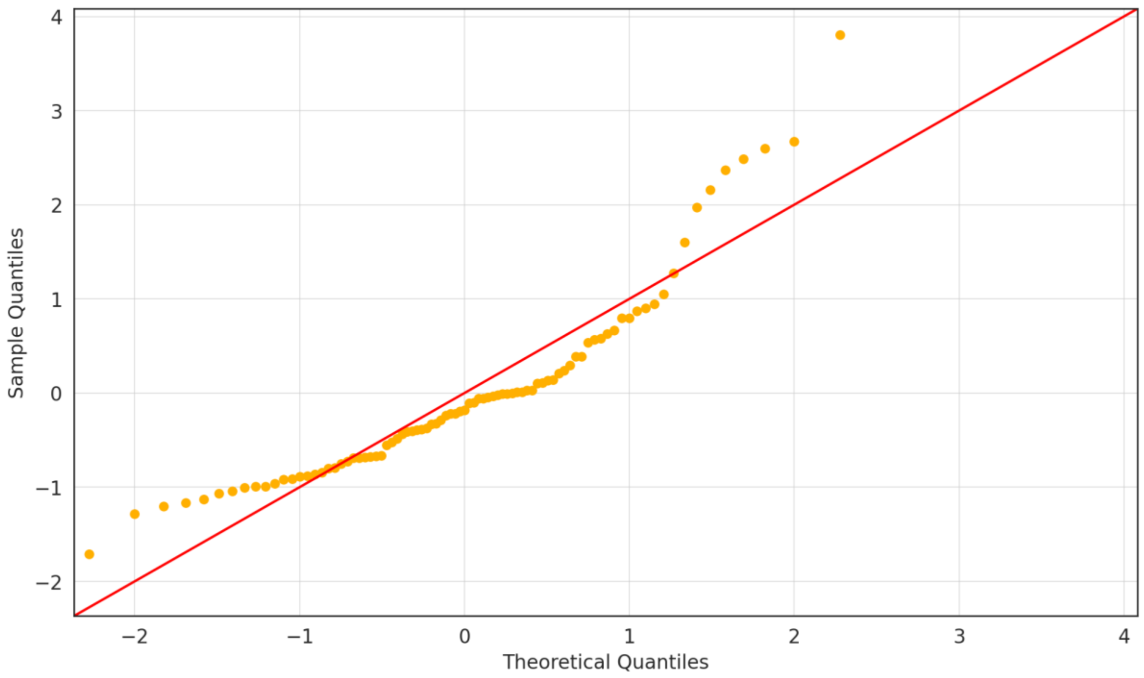

Abbreviations: TMT-B&W-A = trail making test-black and white part A**;** Base Model = No covariates.

Note: The Q-Q plot shows some deviation from the reference line in the upper tail, indicating mild non-normality of residuals. However, linear regression is generally robust to such deviations, especially with a sample size of 87.

Supplementary Figure S3. Residuals vs. Fitted Plot for TMT-B&W-B (Base Model).

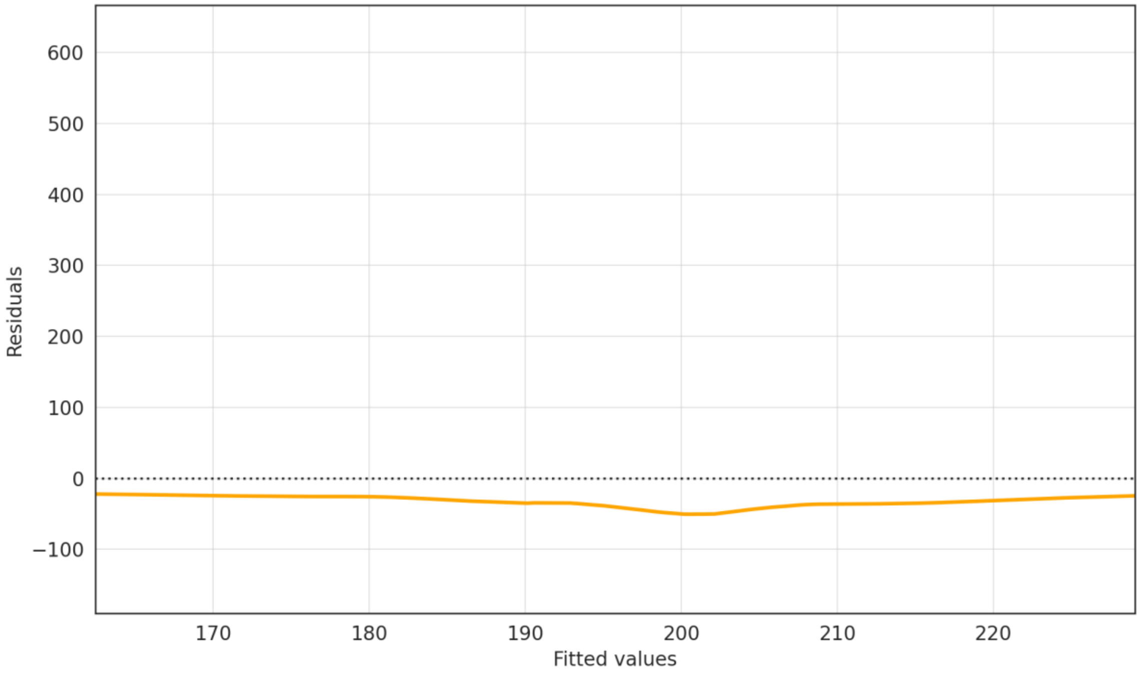

Abbreviations: TMT-B&W-B = trail making test-black and white part B**;** Base Model = No covariates.

Note: The residuals vs. fitted plot shows no curvature or funnel pattern, indicating that the assumptions of linearity and homoscedasticity are reasonably met. This supports the appropriateness of using linear regression for the TMT-B&W-B model.

Supplementary Figure S4. Q-Q Plot of Residuals for TMT-B&W-B (Base Model).

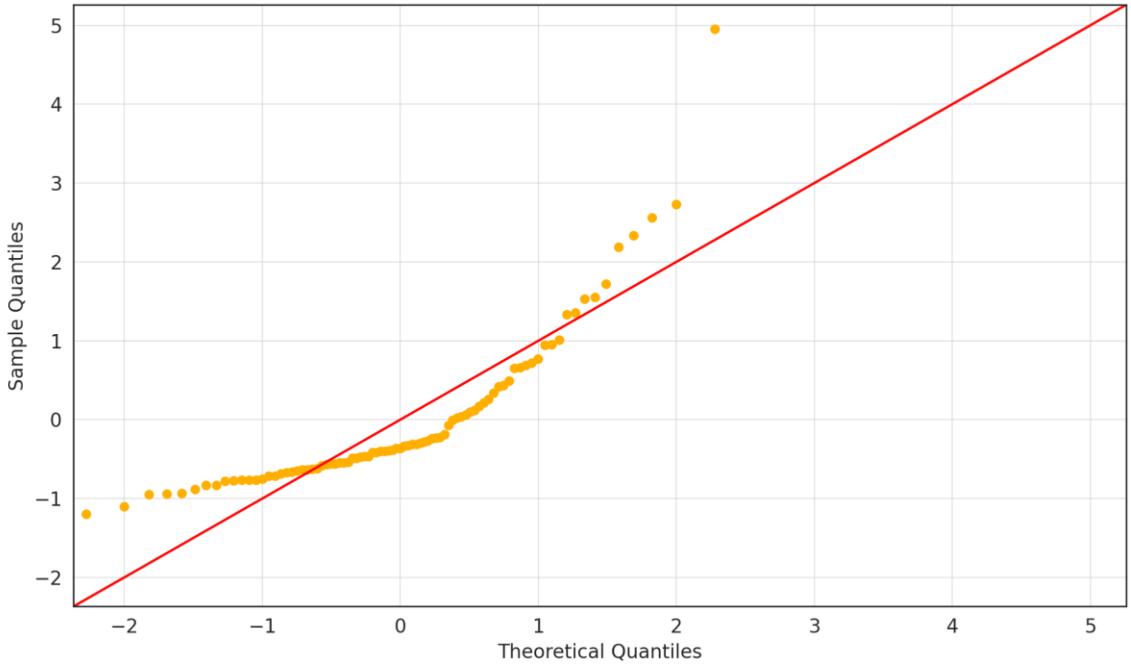

Abbreviations: TMT-B&W-B = trail making test-black and white part B**;** Base Model = No covariates.

Note: The Q-Q plot shows some deviation from the reference line in the upper tail, indicating mild non-normality of residuals. However, linear regression is generally robust to such deviations, especially with a sample size of 87.

Supplementary Figure S5. Residuals vs. Fitted Plot for TMT-B&W-A (Adjusted Model).

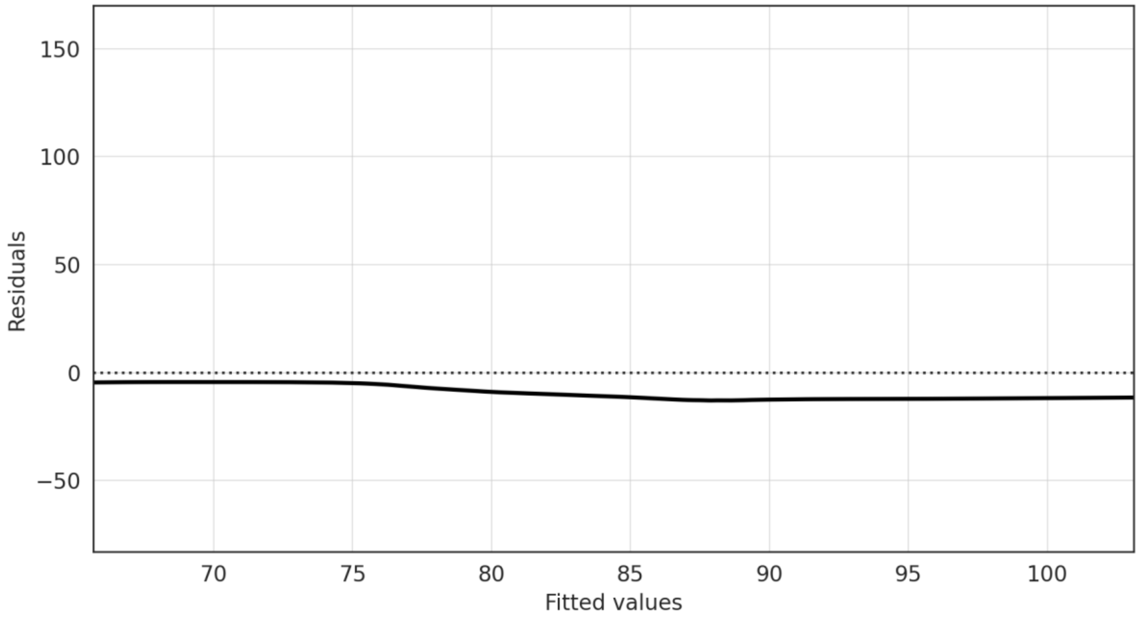

Abbreviations: TMT-B&W-A = trail-making test-black and white part A**;** TICV **=** total intracranial volume; Adjusted Model = age, gender, education, TICV.

Note: The residuals vs. fitted plot shows no curvature or funnel pattern, indicating that the assumptions of linearity and homoscedasticity are reasonably met. This supports the appropriateness of using linear regression for the TMT-B&W-A model.

Supplementary Figure S6. Q-Q Plot of Residuals for TMT-B&W-A (Adjusted Model).

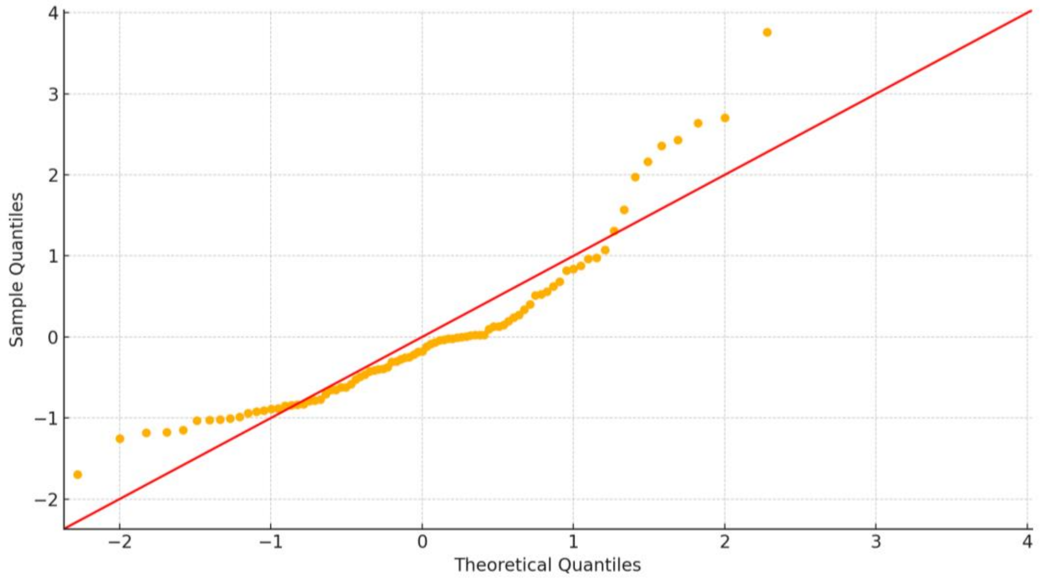

Abbreviations: TMT-B&W-A = trail-making test-black and white part A**;** TICV **=** total intracranial volume; Adjusted Model = age, gender, education, TICV.

Note: The Q-Q plot shows some deviation from the reference line in the upper tail, indicating mild non-normality of residuals. However, linear regression is generally robust to such deviations, especially with a sample size of 87.

Supplementary Figure S7. Residuals vs. Fitted Plot for TMT-B&W-B (Adjusted Model).

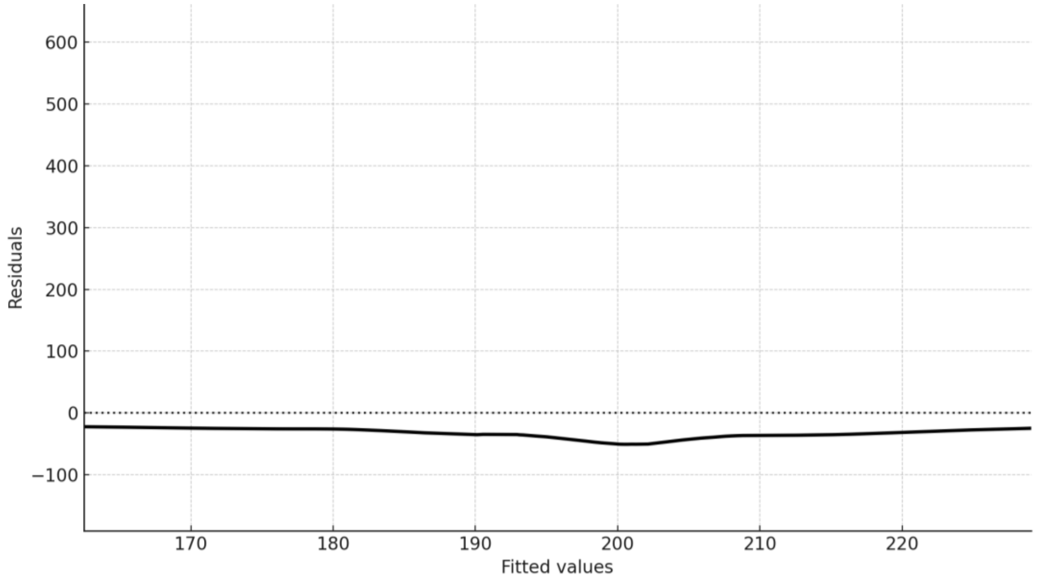

Abbreviations: TMT-B&W-B = trail-making test-black and white part B**;** TICV **=** total intracranial volume; Adjusted Model = age, gender, education, TICV.

Note: The residuals vs. fitted plot shows no curvature or funnel pattern, indicating that the assumptions of linearity and homoscedasticity are reasonably met. This supports the appropriateness of using linear regression for the TMT-B&W-B model.

Supplementary Figure S8. Q-Q Plot of Residuals for TMT-B&W-B (Adjusted Model).

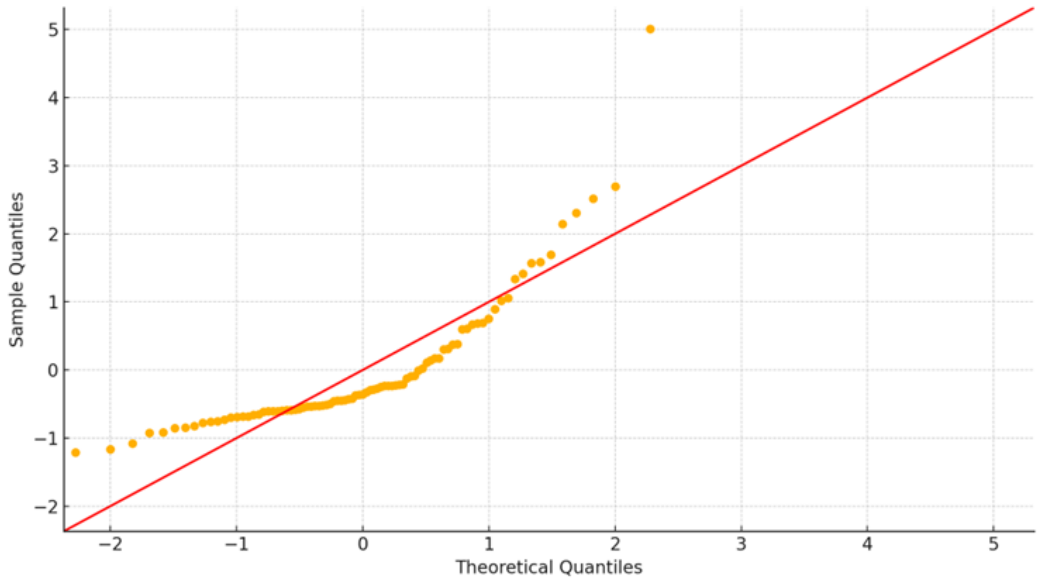

Abbreviations: TMT-B&W-B = trail-making test-black and white part B**;** TICV **=** total intracranial volume; Adjusted Model = age, gender, education, TICV.

Note: The Q-Q plot shows some deviation from the reference line in the upper tail, indicating mild non-normality of residuals. However, linear regression is generally robust to such deviations, especially with a sample size of 87.
